# Management of Acute Appendicitis during COVID-19 Pandemic in a Tertiary Care Centre: A Descriptive Cross-sectional Study

**DOI:** 10.31729/jnma.6307

**Published:** 2021-03-31

**Authors:** Anupama Thapa Basnet, Suraj Singh, Bijay Thapa, Anuj Kayastha

**Affiliations:** 1 Department of Pediatric Surgery, Kanti Children's Hospital, Maharajganj, Kathmandu, Nepal

**Keywords:** *acute appendicitis*, *antibiotics*, *appendectomy*, *COVID-19*, *non-operative management*

## Abstract

**Introduction:**

Acute appendicitis is the most common reason for abdominal surgery in children. Though appendectomy considered the gold standard there are a lot of complications related to it. Conservative management of acute appendicitis is not new to medical science. In pandemic like COVID-19 when all the health systems were about to shut-down because of lack of manpower and resources, we started a trial of non-operative management. The main aim of this study is to find out the management of acute appendicitis during COVID-19.

**Methods:**

This was a descriptive cross-sectional study conducted in a tertiary care centre. Data collection was done from the record section which included the patients diagnosed with acute appendicitis from February 2020 to July 2020 after obtaining ethical clearance from Institutional Review Committee. Cases of appendicular lump, appendicular abscess, appendicular perforations have been excluded. Data was collected and entry was done in Statistical Package for the Social Science software version 20, point estimate at 95% Confidence Interval was calculated along with frequency and proportion for binary data.

**Results:**

The conservative management of acute appendicitis was done in 44 cases (55.7%) while 35 cases (44.3%) had to undergo appendectomy.

**Conclusions:**

COVID-19 can complicate the perioperative course as a result of direct lung injury and multiple organ dysfunctions and can also bring serious threats to the safety of medical staffs involved in managing the acute appendicitis case operatively, so conservative management can be considered as an alternative way of management of acute appendicitis in the pandemic outbreak.

## INTRODUCTION

Acute appendicitis (AA) is the leading reason for abdominal surgery in children.^1-3^ Based upon assumption that AA is irreversible progressive disease, leading to perforation of appendix with subsequent peritonitis, appendectomy is considered as gold standard.^4^ Although curative, appendectomy is invasive procedure requiring general anesthesia with associated perioperative risks and postoperative complications in about 10% within 30 postoperative days.^5,6^

Non-operative management (NOM) of AA, with multiple reported case series of successful treatment of soldiers during wartime or submariners during long missions.^7^ During COVID-19 outbreak, there has been a renewed interest in managing patients with AA nonoperatively with antibiotics, thereby reducing the amount of theatre time, staff time and surgical resources.^1,7,8^ Also, patients with the COVID-19 have substantially higher operative risks due to the compromisation of lung function and the cytokine storms that result in systemic inflammatory response syndrome (SIRS) and multiple organ dysfunction.^9^

The main aim of this study is to find out the management of acute appendicitis during COVID-19.

## METHODS

This was a descriptive cross-sectional study conducted in Kanti Children's Hospital. Ethical clearance was obtained from Institutional review committee - KCH. Data of the patients diagnosed as AA from February 2020 to July 2020 was collected and the age, sex, days of hospitalization, total leukocyte count, lumen diameter, management process, symptoms and signs were analyzed. Cases of appendicular lump, appendicular abscess, appendicular perforation have been excluded. Those patients who didn't respond to antibiotics i.e. no decrease in MANTRELS score from time of admission within 24 hours were operated and the follow-up data of the case undergoing NOM was also analyzed. Sample size was calculated using the formula;

$$\begin{array}{ll} \text{n} & ={\text{Z}}^{\text{2}}\times \text{p}\times \text{q}/{\text{e}}^{\text{2}}\\ & ={\left(1.96\right)}^{2}\times (0.5)\times (1-0.5)/{\left(0.11\right)}^{\text{2}}\\ & =79 \end{array}$$

Where,

n = required sample size,
Z = 1.96 at 95% Confidence Interval
p = prevalence of conservative management in patients of acute appendicitis, 50%
q = 1-p
e = margin of error, 11%

As a part of NOM, patients were kept nil per oral and supplemented with intravenous fluids in maintenance dose, intravenous antibiotics cefotaxime 50 mg/kg body weight/dose thrice daily, metronidazole 7.5-15mg/kg body weight/dose thrice daily, tobramycin 2-2.5mg/kg body weight/dose twice daily, intravenous analgesic ketorolac 0.5mg/kg body weight/dose thrice daily. The antibiotic cefotaxime was upgraded within 24-48 hours of its 1^st^ dose, to Piperacillin + Tazobactam combination 90 mg/kg body weight/dose thrice daily in the case in which there is only minimal subsidence of signs and symptoms, i.e. only decrease in MANTRELS score by 1-3 points from the score at the time of admission. However, decrease in score by ≥4 points were continued the same antibiotics.

The data was entered in MS excel program 2013 and necessary descriptive statistics were obtained using SPSS version 20.

## RESULTS

**Figure 1.**
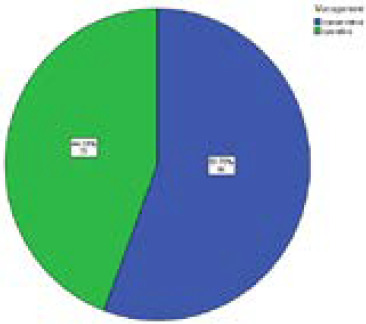
Distribution of management of acute appendicitis.

Among seventy-nine cases of AA 44 cases (55.7%) were managed conservatively, and 35 cases (44.3%) undergone appendectomy because of persistence of signs and symptoms of same intensity as presented to emergency department even after supplemented with the IV antibiotics, IV analgesics and i.v fluid within 24 hours (Figure 1).

**Figure 2.**
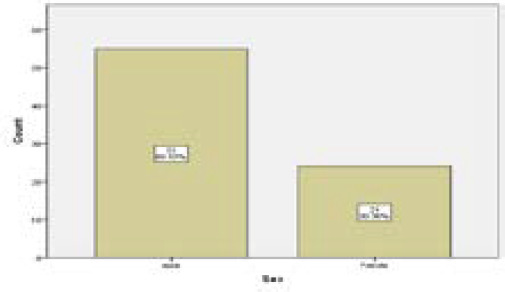
Gender wise distribution of acute appendicitis.

Among the 79 cases of AA, 55 cases (70%) were male, and 24 cases (30%) were female (Figure 2).

The mean age of the patients of AA was found to be 8.81±3.38 years and the mean of white blood cell count in patients of AA was found to be 14140.25±2333.33 cells/mm^3^. Abdominal sonography was done in all cases but the appendix was not visualized in 33 cases and in the remaining 46 cases the mean lumen size was 7.97±2.09 cm. The mean days of hospitalization of patients of AA were found to be 4.46±1.89 days (Table 1).

**Table 1:** Mean and standard deviation of age, total leukocyte count, USG lumen size and days of hospitalization of a patient with acute appendicitis.

	Minimum	Maximum	Mean±SD
Age of Patients (n=79)	1	14	8.81±3.383
Total Leukocyte Count (n=79)	9600	20400	14140.25±2333.327
USG Lumen Size (n=46)	6	19	7.97±2.090
Days of Hospitalization (n=79)	1	15	4.46±1.893

The most common clinical presentation of patients with AA was found to be a pain in the right iliac fossa 79 (100%), followed by nausea 78 (98.7%) and vomiting 61 (77.2%). Fever and rebound tenderness was seen in only 37 (46.8%) and 33 (41.8%) respectively (Table 2).

**Table 2:** Clinical features with their percentage.

Clinical features	n (%)
Pain in RIF	79 (100)
Nausea	78 (98.7)
Vomiting	61 (77.2)
Fever	37 (46.8)
Rebound tenderness	33 (41.8)

The mean length of hospital stay with NOM and operative management of AA was found to be almost equal i.e. 4.41 days with SD of 1.756 and 4.51 days with SD of 2.077 respectively (Table 3).

**Table 3:** Management process with mean days of hospitalization.

Management	Mean±SD (Days)
Conservative	4.41±1.756
Operative	4.51±2.077
Total	4.46±1.893

## DISCUSSION

NOM of AA in the pediatric age group is not new. The impressive success rate of antibiotic treatment has caused a surge of interest in healthcare facilities since the beginning of the pandemic. We have not approved NOM as routine management for AA in our institution but because of the perioperative risks of COVID-19 and cross-infections, we have initiated NOM.

Study done by Georgiou, et al. reported outcomes in 658 children of whom 305 (46%) received NOM and 353 (54%) primary appendectomy.^2^ Study done by Poudel et al reported 11 (11.5%) required appendectomy whereas 79 (82.3%) undergo successful conservative management.^8^ Recently Suwanwongse et al. reported a case report of acute appendicitis in COVID-19 patients with successful treatment with antibiotics.^9^ Early diagnosis and treatment can reduce complications. Once the diagnosis is confirmed, appendectomy is performed either open or laparoscopic.^10,11^ In recent years, surgical removal of the appendix for AA has been reevaluated and NOM is considered a safe and effective way of treatment.^5,12,13^ In a study done by Tanaka, et al. the success rate of nonoperative treatment was 98.7%.^13^ Likewise study done by Hall, et al. total of 1430 participants out of which 727 (50.83%) undergone antibiotic therapy and 703 (49.17%) undergone.^14^

In our study, out of seventy-nine cases of AA 44 cases (55.7%) were managed conservatively, and 35 cases (44.3%) undergone appendectomy. And this shows that percentage of patient treated conservatively was more than the results of study done by Georgiou, et al. and Hall, et al. However the percentage of patient undergoing conservative management is less in comparison to result of study done by Poudel et al.

NOM of AA in children is acceptable in both complicated and uncomplicated cases.^20^

Antibiotic therapy has an important role in the management of AA. Antibiotics that cover enteric gram negative organisms, anaerobes, E coli, and Bacteroides species should be started as soon as the diagnosis of appendicitis.^21^ Antibiotic therapy should be continued for 24 to 72 hours depending upon the condition of the child. The result of inadequate treatment can be very severe. Therefore, treatment should be done in the hospital under the supervision of doctors, and patients can be discharged home on oral antibiotics only after the complete recovery of the patient.^22^

In our study, we used cefotaxime, tobramycin, and metronidazole. If the patient's condition were improving and were tolerating oral, then we had switched to oral antibiotics and discharged the patient. But if the patient was improving partially within 24 to 48 hours, then we had counselled the patient party before upgrading antibiotics to piperacillin and tazobactam combination and observing the patient. If the child's condition was not improving at all, then we proceeded with appendectomy.

NOM for AA is less invasive and has less morbidity related with surgery and anesthesia, but is associated with failure of management and the risks of relapses. Study done by Mudri et al. shows that recurrence rate of NOM of AA is 21%, during one year follow up.^25^ In our study, the recurrence rate for NOM is difficult to assess due to quite short follow up i.e. 2^nd^ and 6^th^ week of discharge.

There are no as such studies that describe this trend in more detail, so more study need to be done to find out the management in AA during pandemic outbreak. Our study has reflected the case of AA of short time period, i.e. only six months duration. In addition, we have only information that had been documented and accessible in our medical records. Hence our study have some limitations and lacks external validity.

## CONCLUSIONS

COVID-19 patients may have high postoperative mortality, and postoperative respiratory complications. Medical staff serving operative patients is at high risks of cross-infection. Thus, NOM of AA may be an alternative choice of treatment during the pandemic outbreaks like COVID-19. However, an optimal selection of antibiotics and a close observation of the medical person is required. Besides that, the chance of readmission is quite high, which needs further research and long term follow up to decide the wellbeing and benefits of broad spectrum antibiotics versus an appendectomy.
